# Strength Amid Strain: Coping, Racism, and Racial Socialization Stress in Black Caregivers

**DOI:** 10.3390/bs16030326

**Published:** 2026-02-27

**Authors:** Emani Sargent, Marlena Debreaux, Sheretta T. Butler-Barnes, Ivy Smith, JaNiene Peoples

**Affiliations:** 1 Brown School of Social Work, Washington University in St. Louis, St. Louis, MO 63130, USA; sargente@wustl.edu (E.S.); marlena@wustl.edu (M.D.); 2 Division of Computational & Data Sciences, Washington University in St. Louis, St. Louis, MO 63130, USA; ivy.s@wustl.edu; 3 College of Social Work, Florida State University, Tallahassee, FL 32306, USA; jpeoples2@fsu.edu

**Keywords:** racial socialization stress, racism, black caregivers, coping self-efficacy

## Abstract

This study examined Black caregivers’ affective responses to racial discrimination (i.e., the extent to which they reported being bothered by discriminatory experiences) and how these responses were associated with stress associated with navigating racial socialization practices (i.e., stress during conversations about race and racism with children). We further tested whether coping self-efficacy beliefs (i.e., problem-focused coping, suppressing unpleasant emotions and thoughts, and seeking support from family and friends) moderated the association between racial discrimination and racial socialization stress. The sample included a socioeconomically diverse sample of 680 Black caregivers (*M*_age_ = 37, 55% mothers). A significant interaction indicated that among caregivers who reported being highly bothered by racial discrimination, higher levels of problem-focused coping were associated with greater racial socialization stress, whereas lower levels of problem-focused coping were associated with lower stress. Being highly bothered by racial discrimination and reporting high levels of stopping unpleasant emotions and thoughts as a coping strategy was associated with the lower levels of racial socialization stress in comparison to those with lower levels of stopping unpleasant emotions and thoughts. Black caregivers, under the conditions of reporting being bothered by racism, with higher levels of family and friend support had lower levels of racial socialization stress in comparison to those with lower levels of family and friend support. The findings highlight the need to support Black caregivers in building effective coping strategies and social support networks.

## 1. Introduction

Black Americans’ experiences in the United States are deeply rooted in a history marked by systemic oppression, racial discrimination, and prejudice. Despite significant progress made through the civil rights movement, Black Americans continue to face disproportionate barriers in various domains, including housing, employment, education, and the criminal justice system (Banaji et al., 2021; Paige, 2022). Since the Civil Rights and Black Power movements, racial socialization has been understood as a critical intergenerational process through which Black caregivers prepare youth to navigate racism. However, this literature has primarily focused on the content of racial messages rather than the psychological burden caregivers may experience when delivering them. Thus, although the Civil Rights and Black Power movements foregrounded collective resistance and racial pride, contemporary racial socialization messaging often unfolds within the private sphere of family life, where caregivers must translate structural inequities into developmentally appropriate guidance. This shift situates racial socialization not only as cultural transmission, but as emotionally demanding labor. Additionally, the rise in movements like Black Lives Matter is a testament to the ongoing struggle against racial discrimination and the need for societal reform, especially in Missouri. Within this context, Black parents face the complex task of preparing their children to navigate a world that may still harbor biases against them, making racial socialization an essential parenting practice (Anderson & Stevenson, 2019). In the current study, we center racial socialization stress as a distinct construct reflecting the psychological strain caregivers experience when navigating conversations about race and racism. Further, by examining the moderating role of coping self-efficacy, we move beyond documenting stress to examining variability in caregivers perceived capacity to manage stress when having conversations with their children about race and racism. Additionally, racial socialization stress is conceptualized as distinct from both general parenting stress and general racial stress. Whereas racial stress reflects the psychological impact of discriminatory experiences (Utsey & Ponterotto, 1996) and parenting stress (Peterson et al., 2010) captures global caregiving strain, racial socialization stress specifically refers to the burden associated with preparing children to understand and cope with racism (Anderson & Stevenson, 2019).

As previous stated, racial socialization stress, a dimension of racial socialization competency, refers to the psychological and emotional challenges individuals face when navigating and responding to racism, particularly in families or communities (Anderson & Stevenson, 2019). Racial socialization involves transmitting messages about race, identity, and coping with racism, which can be a protective factor against racial stressors (Anderson et al., 2024; S. C. T. Jones et al., 2021a). However, for individuals tasked with teaching or learning these messages, the process can be stressful, especially when facing societal pressures, intergenerational differences, or personal uncertainties about how to address race-related issues (Anderson & Stevenson, 2019; Charity-Parker & Adams-Bass, 2023; Christophe et al., 2022; Jacob et al., 2022). This stress may arise from the difficulty of balancing positive affirmations of racial identity with preparing for discrimination, potentially affecting mental health and the effectiveness of racial socialization efforts (Anderson et al., 2024; Osborne et al., 2020; Wilson & Gentzler, 2021). For Black caregivers, this process of racial socialization represents a critical parenting practice (S. C. T. Jones et al., 2021b). The Racial Encounter Coping Appraisal and Socialization Theory highlights that a caregiver’s skill and confidence in delivering racial socialization messages are crucial for fostering effective coping mechanisms in youth facing discriminatory racial encounters (Anderson & Stevenson, 2019; Galán et al., 2021). This theoretical framework emphasizes the importance of caregiver competence in racial socialization, aligning with cognitive-behavioral approaches that focus on emotional and self-perception processes to mitigate psychological harm from racial discrimination (Anderson et al., 2021; S. C. T. Jones et al., 2023). This includes equipping parents and support systems with the proficiency to articulate racial pride and adeptly guide Black children in managing racialized experiences (Charity-Parker & Adams-Bass, 2023). This competency involves not only the content of the messages but also the perceived skills, confidence, and stress associated with their delivery, all of which contribute to more optimal psychosocial outcomes in adolescents by interfering with the stressful transmission of negative behaviors and moods (Anderson et al., 2021).

In the present study, we investigate the experiences of Black caregivers regarding racial discrimination on their racial socialization stress when engaging in discussions about race and racism with their child. We also explore the moderating role of coping self-efficacy beliefs, which encompass problem-focused coping, suppressing unpleasant emotions and thoughts, and seeking support from family and friends. This inquiry is critical, as existing research indicates that parental experiences with racism significantly influence the efficacy of their racial socialization messages (Osborne et al., 2020).

## 2. Black Americans’ Experiences with Racialized Challenges

Racial distress refers to the emotional and mental harm caused by ongoing exposure to racism and discrimination (Carter, 2006). While it may result from a single traumatic event, it more commonly emerges as the result of cumulative experiences (Comas-Díaz et al., 2019). Black Americans face higher psychological distress due to racism than White Americans (Williams, 2018). For instance, 75% of Black adults report experiencing racism (Pew Research Center, 2024). These inequities are driven primarily by systemic and institutional racism, racial and intergenerational trauma, inequities in treatment, and vicarious exposure to racial violence trauma (Hankerson et al., 2022; Moody, 2022).

Existing literature consistently demonstrates that racial and intergenerational trauma also contribute to increased levels of psychological strain among Black adults. Hankerson et al. (2022) explain that racial intergenerational trauma can be transmitted across generations because of historical and ongoing systemic racism, and when this inherited trauma combines with present-day racial stressors, many Black Americans experience psychological distress, including chronic anxiety, hypervigilance, and depressive symptoms. For instance, Moody (2022) found that indirect exposure to race-based violence can result in heightened distress among Black adults. Collectively, this body of research demonstrates that psychological distress among Black Americans is not isolated nor individualized, but rather the cumulative product of structural, interpersonal, and vicarious racial stressors. These ongoing stressors not only shape Black adults’ affective responses to racial discrimination but also influence caregiving practices and the racial socialization strategies Black caregivers use to prepare their children to navigate racism.

## 3. Racial Socialization Stress Among Black Caregivers

Racial socialization, the process through which parents communicate messages about race, cultural pride, and strategies for managing discrimination, is a vital parenting practice within Black families, yet it represents one of the most psychologically taxing aspects of raising children in a racially stratified society (Anderson & Stevenson, 2019). While substantial research demonstrates that racial socialization serves as a protective factor for youth mental health and resilience, considerably less attention has been directed toward the psychological toll this parenting task places on caregivers themselves. Black caregivers must simultaneously process their own experiences with racial discrimination, manage anxiety about their children’s safety, and deliver messages about both cultural pride and racial threat (Anderson & Stevenson, 2019; Murry et al., 2018). This constellation of demands creates racial socialization stress, the psychological and emotional strain that emerges when caregivers attempt to prepare their children for racism while contending with their own racial distress (Anderson & Stevenson, 2019).

The roots of racial socialization stress lie in a fundamental paradox, caregivers must foster positive racial identity and self-esteem in their children while simultaneously preparing them to navigate a world where racial discrimination poses genuine threats (Anderson & Stevenson, 2019). A longitudinal investigation conducted by Saleem et al. (2025) demonstrated an association between Black parental concerns regarding racial profiling and heightened stress levels experienced during discussions about race. Critically, this worry-related stress extended to greater internalizing symptoms in youth, suggesting that parental emotional dysregulation can be transmitted to children through family interactions (Saleem et al., 2025). Research examining mothers’ responses to publicized incidents of anti-Black violence found that 68% of mothers of Black children predicted negative implications for their own children’s wellbeing (Thomas & Blackmon, 2015).

The stress of racial socialization cannot be disentangled from caregivers’ own ongoing exposure to racial discrimination. Black adults who report higher frequencies of perceived racial discrimination also report elevated levels of stress and psychological distress (Pascoe & Smart Richman, 2009). Research examining how discrimination-related distress influences parenting found that higher levels of multidimensional discrimination distress were associated with more maternal overcontrol, which can undermine the open, supportive communication necessary for effective racial socialization (Kazmierski et al., 2023). Additionally, structural racism creates the conditions that intensify racial socialization stress. Caregivers living under conditions of surveillance systems experience compounded stress that limits their capacity to engage in effective racial socialization (Murry et al., 2018).

Racial socialization competency encompasses three dimensions, confidence (parents’ beliefs in their ability to communicate effectively about race), skills (the proficiencies in delivering racial messages), and stress (the psychological burden associated with the process) (Anderson & Stevenson, 2019). We focus on racial socialization stress in the current study. Mentioned previously, racial socialization stress is an emotionally intense, cognitively demanding process occurring within persistent exposure to racial discrimination (Anderson & Stevenson, 2019; Murry et al., 2018).

## 4. Coping Self-Efficacy: Problem Solving, Emotions, and Social Support

The capacity of Black caregivers to parent effectively in racially adverse environments depends not only on the presence of discrimination but also on their belief in their ability to manage stress. Coping self-efficacy, defined as confidence in one’s ability to handle stressful events (Bandura, 1997), is a key factor in understanding how individuals remain psychologically resilient when facing stressful situations. Drawing from social cognitive theory and health behavior models, self-efficacy is understood to influence whether individuals adopt adaptive coping methods, remain engaged during hardship, and recover from emotional strain (Glanz et al., 2008).

Coping self-efficacy is closely tied to stress appraisal. Notably, parental worries and emotional states can influence perceived efficacy. Caregivers who believe they can manage distressing thoughts, use problem-solving strategies, and lean on others for support may experience less emotional strain when engaging in racially charged parenting tasks such as racial socialization (Murry et al., 2018; Smith et al., 2022). Coping self-efficacy is understood as multidimensional, encompassing three domains that include problem-focused coping (e.g., regulating responses in challenging situations), stopping unpleasant thoughts and emotions (e.g., managing unpleasant feelings or thoughts), and family and friend support (e.g., support from family and friends when facing stress) (Chesney et al., 2006). These domains are particularly relevant when examining caregiver stress due to racism.

Problem-focused coping is often discussed as a dimension associated with favorable outcomes, yet in situations where racism is deeply embedded and difficult to change, these strategies can sometimes lead to frustration or a sense of ineffectiveness (Compas et al., 2001). In contrast, emotion-focused approaches such as reframing or controlling intrusive thoughts may help caregivers regulate their own responses and stay emotionally available during difficult discussions with children (Compas et al., 2001). These strategies are especially important for caregivers who must process their own experiences of racial discrimination while guiding their children through similar challenges (Anderson et al., 2021).

Alongside internal coping beliefs, support from others plays an important role in shaping how caregivers respond to racial stress and engage in racial socialization. Social support refers to the perceived availability of emotional, informational, or practical help from others (Thoits, 2011). For many Black caregivers, support networks may include chosen and extended family, friends, faith communities, and civic or cultural organizations. These networks can offer both emotional reassurance and a shared understanding of racial stress, while also reinforcing culturally grounded approaches to parenting (Murry et al., 2018; Saleem et al., 2016).

Black parents who report less stress in preparing their children to recognize and respond to racism are also more likely to experience reduced psychological strain when confronted with racial stressors (Brooks Stephens et al., 2023). Racial socialization stress appears to shape not only how parents engage in racial socialization but also how they manage their own emotional responses. In contexts marked by persistent racial threat, caregivers who feel equipped to communicate effectively may be better able to remain emotionally present for their children. Social support may further reinforce this capacity. As described by Wills and Shinar (2000), support from others can interrupt the link between acute and chronic stress. This pattern is particularly relevant for Black families, who often contend with the compounded demands of personal, cultural, and structural racism. Evidence suggests that when caregivers can rely on both internal resources and external support, they may be better positioned to carry out the complex emotional labor of racial socialization.

Even so, few studies have examined how coping self-efficacy (i.e., problem-focused coping, stopping unpleasant thoughts and emotions, and family and friend support) serves as a moderator between racial discrimination and racial socialization stress. Much of the existing literature has focused on youth outcomes. Given that racial socialization can itself be a source of stress for caregivers (Anderson et al., 2021; Brooks Stephens et al., 2023), it is important to understand how coping self-efficacy shapes this process.

Coping is a crucial component of parenting for Black caregivers that influences how they manage their own affective responses to racial discrimination (i.e., the extent to which they report being bothered by discriminatory experiences) and how they equip their children. When parents have high coping self-efficacy, they might be more likely to confront racism proactively, maintain emotional equilibrium, and model effective coping for their children. When they are bothered by racial discrimination, they may either avoid the topic or convey a sense of pessimism, which can leave children less prepared. Recognizing this, the current literature is increasingly focusing on empowering parents with coping skills and affirming their capacity to foster resilience.

## 5. Guiding Framework

This study draws on Murry et al.’s (2018) Sociohistorical Integrative Model for the Study of Stress in Black Families. The Sociohistorical Integrative Model for the Study of Stress in Black Families offers a culturally informed model for understanding Black family stress and adaptation. This approach foregrounds racism as a routine feature of daily life for Black families, rather than a rare or isolated occurrence. It also emphasizes the importance of community ties, cultural strengths, and context-specific strategies in shaping how families respond to stress. In the present study, we specifically investigate parents’ reports of affective responses to racial discrimination (i.e., the extent to which they reported being bothered by discriminatory experiences) and the impact on racial socialization stress. Although the Sociohistorical Integrative Model highlights the racial stress and adaptive coping within Black families, it does not specify how coping strategies may shape caregivers’ experiences of racial socialization stress. Importantly, coping strategies do not operate in a vacuum. Within sociohistorical contexts that are characterized by racism, coping strategies may vary. As such, the moderating role of coping self-efficacy beliefs will be examined. Using problem-focused strategies, stopping unpleasant emotions and thoughts, and seeking support from family and friends are all examples of coping mechanisms that might help to mitigate the pervasive effects of racial stressors (Cooper et al., 2020), reducing the stress from parents’ racially discriminatory experiences, thereby fostering greater well-being and adaptive parenting (Anderson et al., 2018b, 2021; Anderson & Stevenson, 2019). More specifically, parental racial discrimination experiences can lead to worry, which in turn affects their racial socialization competency (i.e., racial socialization stress) (Anderson et al., 2021). This framework further allows for the examination of how coping self-efficacy can reduce the negative impact of being bothered by discrimination on racial socialization stress.

## 6. Current Study

In the current study, we build on the previous literature by examining Black parents reports of affective responses to racial discrimination (i.e., the extent to which they reported being bothered by discriminatory experiences) and how these responses were associated with stress associated with navigating racial socialization practices (i.e., stress during conversations about race and racism with children). We further tested whether coping self-efficacy beliefs (i.e., problem-focused coping, suppressing unpleasant emotions and thoughts, and seeking support from family and friends) moderated the association between racial discrimination and racial socialization stress. Specifically, we investigate how parents’ personal experiences with racial discrimination influence their perceived stress during racial socialization discussions, and how their use of coping self-efficacy beliefs may mitigate that stress. Based on the previous literature documenting the link between racial discrimination and racial socialization stress, we hypothesize that increased experiences of racial discrimination will be associated with higher racial socialization stress (Anderson et al., 2018b). We further hypothesize that coping self-efficacy will moderate the association; however, given the limited research differentiating coping strategies in this context, we do not propose directional predictions regarding which coping strategies would be most protective. This investigation seeks to delineate the intricate interplay between parental lived experiences of racial discrimination and their subsequent engagement in racial socialization stress and the ameliorative potential of coping self-efficacy (Anderson & Stevenson, 2019).

### Positionality Statement

The authors of this study are Black scholars whose social identities and experiences are shaped being raised in the U.S. South and Midwest. Our lived experiences within racially stratified contexts inform our scholarly engagement from interdisciplinary training in developmental science, community psychology, social work, and data and computational sciences. We approach this work from interdisciplinary training with a focus on racism and its implication for Black American families. Collectively, our positionality shapes the questions we ask and the interpretations we consider, particularly regarding the emotional labor and complexity of racial discrimination and racial socialization strategies. At the same time, our methodological training emphasizes statistical rigor, cautious interpretation, and attentiveness to alternative explanations that challenge our own biases. We center Black caregivers as knowledge holders and experts navigating environments, while being mindful that our own experiences inevitably influence the framing and meaning making of this research.

**Figure 1:**
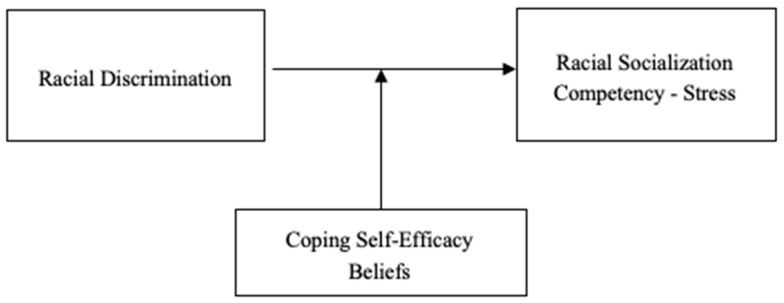
Conceptual Model.

In this study, race refers to socially constructed categories (for example, Black/African American) and the meanings attached to them that shape identity and lived experience in the United States (J. M. Jones, 1997). These categories are also institutionalized through measurement systems such as the U.S. Census, which has repeatedly changed racial classification practices over time, highlighting race as a fluid social and political construct rather than a fixed biological factor (Hirschman et al., 2000; Saperstein & Penner, 2012; Ventura & Flores, 2025). Racism, by contrast, refers to the multilevel system of dynamic forces (individual, institutional and cultural) that produce and sustain social power, a race hierarchy and unequal exposure to harm (J. M. Jones, 1997). Within this framework, at the family level, racial discrimination reflects the interpersonal encounters of racism that caregivers experience in daily life, and these encounters can accumulate into race-related distress and parenting strain (Anderson & Stevenson, 2019; Carter, 2006; Pascoe & Smart Richman, 2009). In this context, conversations about race might emphasize communicating cultural pride and positive racial identity, while conversations about racism might emphasize coping with discrimination (such as preparation for bias or promotion of mistrust) (Anderson & Stevenson, 2019; Hughes et al., 2006; Lesane-Brown, 2006). This distinction is central to the current study as caregivers’ reports of Black caregivers’ affective responses to racial discrimination (i.e., the extent to which they reported being bothered by discriminatory experiences), while racial socialization stress reflects the emotional labor of having tough conversations and preparing children to navigate racism within a racially stratified society (Anderson & Stevenson, 2019; Murry et al., 2018) (see Figure 1).

## 7. Method

The current study is part of an National Science Foundation (NSF) larger study, the (study masked) conducted by masked authors. The study is a community-based, three-year longitudinal study, starting with the first wave of approximately 682 Black families in the state of Missouri in 2022. The families were primarily from urban and suburban communities in Missouri during the sociopolitical climate following the murder of George Floyd. This period was characterized by heightened national and regional racial discourse. The study is unique in several respects: The study is unique in several respects: It includes a sizable proportion of Black families from geographically diverse (i.e., rural and urban) areas in the state. The sample was also socioeconomically diverse and included a range of income and family education levels.

### 7.1. Participants

The present study examines the first wave of the BFRJS data set. A total of 682 families participated in Wave 1 of the study. The final sample for the present study included 680 parents (*M*_age_ = 37, 55% mothers) only with complete data for all the study variables. The percentage of missing data was 1%. In total 680 families out of 682 provided complete data for inclusion in the analyses. A total of 98.4% of parents identified as African American, 1.3% identified as African, and 1 identified as biracial/multiracial, and 1 had missing information. The median household income level for the sample parents was on average, $35,000 to $50,000 per year and on average, indicated that parents completed an associate degree or some college (*M* = 4.29, *SD* = 0.69). Fifty-five percent of the sample were mothers.

### 7.2. Procedure

For the current study, wave one of Black parents completed surveys either online or in person. The surveys took approximately 45 min to one hour to complete, including demographic and individual surveys about their own racialized experiences and parenting. Parents provided informed consent. The Institutional Review Board (IRB) approved this study (#202112032). Parents were recruited over a six-month timeframe. Fliers were distributed to places of worship, school districts, family agencies and organizations, Black businesses (e.g., beauty salons, barber shops, and restaurants), National Panhellenic Organizations (e.g., Divine 9), the city’s housing authority, and at local festivals (e.g., Juneteenth and African). Fliers provided information about the three-year study and contact information to address any questions or concerns about participation in the study. Parents followed the link on the flier, provided consent to participate, and completed the surveys online. Hard copies of the survey were provided for families upon request. The fliers were strategically placed in prominent locations within the community to maximize visibility and reach. Additionally, community leaders were engaged to help spread the word about the study and encourage participation among families.

### 7.3. Measures

Demographics: Caregivers were asked to provide a range of socio-demographic information. They reported factors such as their age and gender, with all participants identifying as either male or female. Caregivers also indicated their relationship to the child, selecting either mother/stepmother or father/stepfather. Additionally, the number of children in the household was an open-ended item, and caregivers shared their educational level.

#### Racial Discrimination

Direct Racism: The Racism and Life Experiences Scale (RaLES) was used to assess parents’ experiences with racial discrimination (Harrell, 1997). There are two subscales: the *frequency* of racial discrimination experiences and how often individuals are *bothered* by racial discrimination. For the current study, the appraisal-based “being bothered” subscale, which captures caregivers’ emotional responses to discriminatory experiences rather than exposure frequency was used. The scale taps into how often one is bothered by their encounters of racial discrimination and ranges from (0 = never happened to me) to (6 = bothered me extremely). There is a total of 18 racial hassles (e.g., in the past year, how much did it bother you …“ignored or overlooked,” or “treated suspiciously in a store”?) In previous research (e.g., Richardson et al., 2015), the alpha values ranged from 0.85 to 0.90. The items were summed and averaged across the 18-items. The alpha for the bothered by racial discrimination is 0.92.

- *Moderators*

Coping Self-Efficacy: The Coping Self-Efficacy Scale assesses perceived self-efficacy in coping with life’s demands and stressors (Chesney et al., 2006). The scale measures three areas: the use of problem-focused coping strategies, stopping unpleasant emotions and thoughts, and access to family support and resources. The scale comprises 26 items and ranges from 0 = cannot do at all to 10 = certain can do (e.g., sort out what can be changed, and what cannot be changed). The three subscales were used and the alphas for problem-focused coping, stopping unpleasant thoughts and emotions, and support family and friends were 0.77, 0.76, and 0.66, respectively.

- *Socialization Outcome*

Racial Socialization Stress: The Racial Socialization Competency Scale (Anderson & Stevenson, 2019) was used to assess parents’ racial socialization competencies. There are three subscales: confidence, skills, and stress. The scale is 28-items. In this study, we used the *stress subscale* to capture caregivers’ racial socialization stress. Items from the stress subscale were summed and averaged across the 28 items, with higher scores reflecting greater stress. Parents were given the prompt: stress (e.g., “I am/would be stressed to share my emotions about my experiences of negative racial encounters.”). Responses ranged from 1 = very unstressed to 5 = very stressed. In the current sample, the alpha is 0.94.

### 7.4. Data Analyses Plan

SPSSS 29.0 was used to analyze the data. Hierarchical regression was used to test moderation effects. Covariates (age, education, number of children in the household, income, and caregiver gender) were entered in Step 1 to account for demographic influences. Racial discrimination was entered in Step 2 to estimate the main effect. Coping self-efficacy (i.e., problem-focused coping strategies, stopping unpleasant emotions and thoughts, and friend and family support) were entered in Step 3 to assess their independent associations. Lastly, interaction effects were entered in Step 4 to test moderation effects. This block ordering follows recommended procedures for testing interaction effects in regression (Aiken & West, 1991).

## 8. Results

Means, standard deviations, and correlations for the primary study variables are presented in Table 1. Parents reported on average receiving an Associate’s degree or attending some college/university (4-year college or university) and earning $35,000 to $50,000 per year. On average, parents had two children. Parents reported being bothered by racial discrimination. In addition, parents expressed varying degrees of coping self-efficacy strategies (see Table 1).

Correlations. There were statistically significant correlations between the independent, moderation, and outcome variables (see Table 2). Higher levels of being bothered by racial discrimination was correlated with higher levels of coping self-efficacy strategies. Being bothered by racial discrimination was also correlated with lower racial socialization stress.

### Racial Socialization Stress

The outcome variable in the model was racial socialization stress. In block 1, age, education, number of children, household income, and gender. In block 2, bothered by racial discrimination was entered. In block 3, coping self-efficacy strategies, problem-focused coping, stopping unpleasant thoughts and emotions, and support from family and friends (moderators), were entered, and the three interaction terms were entered in block 4: racial discrimination × problem-focused coping, racial discrimination × stopping unpleasant thoughts and emotions, and racial discrimination × support from family and friends. The variables were centered before being entered into regression models, as advised by Aiken and West (1991), when testing interaction effects. Significant interaction effects were examined by plotting the slope of racial socialization stress regressed on coping self-efficacy strategies estimated at selected conditional values (*M* + 1 *SD* and *M* − 1 *SD*) of being bothered by racial discrimination (Cohen & Cohen, 1983).

Overall, the results showed that the first model was significant *F*(5,674) = 68.39, *p* = 0.001, *R*^2^ *=* 0.33. Caregivers’ education (*β* = −0.42, *p* = 0.001), number of children in the household (*β* = 0.26, *p* = 0.001), household income (*β* = 0.16, *p* = 0.001) were statistically significant. The findings revealed that higher education levels were associated with less racial socialization stress. Parents who also reported more children and higher income were associated with higher levels of stress. The second model (*F*(6,673) = 110.11, *p* < 0.001, *R*^2^ = 0.49), which included being bothered by racial discrimination (*β* = −0.49, *p* = 0.001) showed significant improvement from the first model ∆*F*(1,673) = 211.75, *p* < 0.001, ∆*R*^2^ = 0.16. Parents who reported higher levels of being bothered by racial discrimination reported lower levels of being stressed about having conversations about race and racism. In the third model, problem-focused coping (*β* = −0.06, *p* = 0.130), stopping unpleasant thoughts and emotions (*β* = −0.12, *p* = 0.005), and support from family and friends (*β* = −0.08, *p* = 0.029) were added to the model. The model continued to significantly predict racial socialization stress (∆*F*(3,670) = 25.78, *p* < 0.001, ∆*R*^2^ = 0.05). Overall, higher levels of stopping unpleasant thoughts and emotions and support from family and friends were associated with lower levels of racial socialization stress.

**Figure 2:**
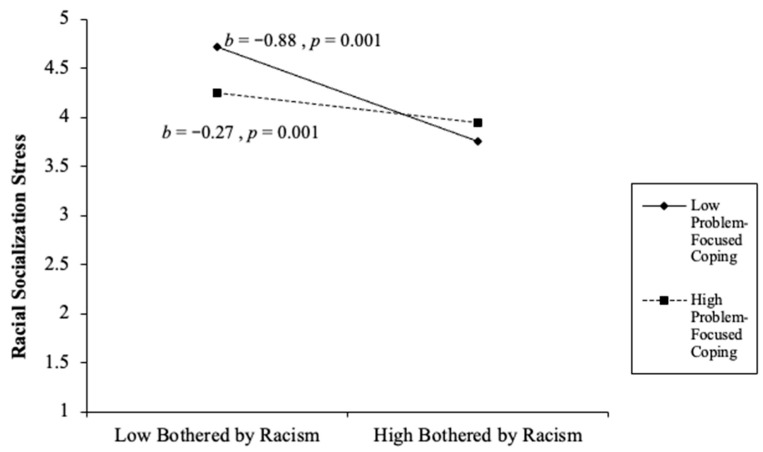
Racism × Problem-Focused Coping Interaction Effect.

**Figure 3:**
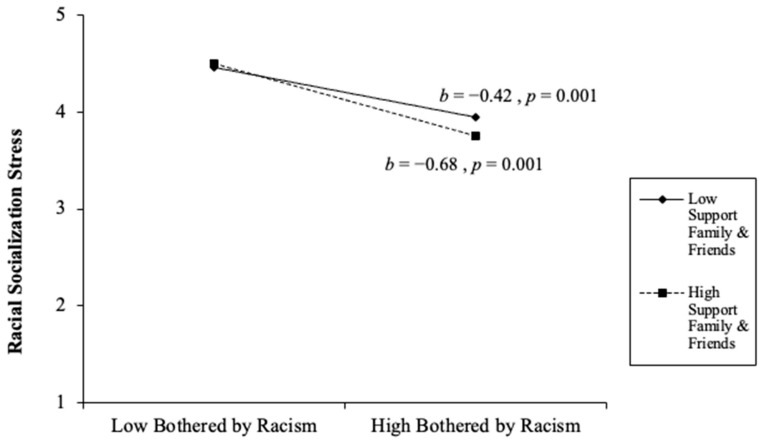
Racism × Support from Family and Friends Interaction Effect.

**Figure 4:**
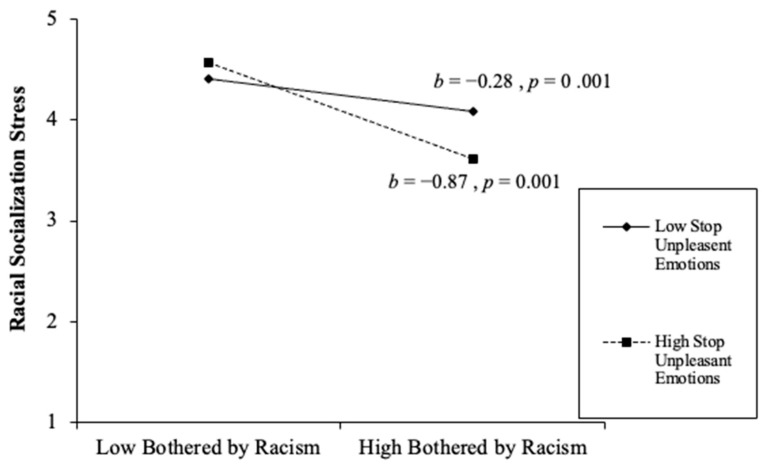
Racism × Stopping Unpleasant Emotions and Thoughts Interaction Effect.

Overall, the final model continued to predict racial socialization stress. The final step involved the addition of three interaction effects: racial discrimination × problem-focused coping, racial discrimination × stopping unpleasant thoughts and emotions, and racial discrimination × support from family and friends. All three interaction effects were statistically significant. The racial discrimination × problem-focused coping significantly predicted caregivers’ racial socialization stress (*β* = 0.23, *p* = 0.001). The racial discrimination × support from family and friends significantly predicted caregivers’ racial socialization stress (*β* = −0.07, *p* = 0.026). The racial discrimination × stopping unpleasant thoughts and emotions significantly predicted caregivers’ racial socialization competency stress (*β* = −0.20, *p* = 0.001). Overall, the final model accounted for 27% of the variance (∆*F*(3,667) = 14.49, *p* < 0.001, ∆*R*^2^ = 0.03) (see Table 3 and Figure 2, Figure 3 and Figure 4). In the racial discrimination × problem-focused coping, parents with low problem-focused coping reported higher racial socialization stress when being bothered by racism was low, but substantially lower stress when being bothered by racism was high. For parents with high problem-focused coping, racial socialization stress remained relatively stable across levels of being bothered by racism. Together, these findings suggest that problem-focused coping moderates the relationship between racism-related stress and racial socialization stress, such that differences in stress by being bothered by racism are more pronounced among parents with lower problem-focused coping (see Figure 2). As depicted in Figure 3, parents with higher support from family and friends reported slightly higher racial socialization stress when being bothered by racism was low, but substantially lower stress when being bothered by racism was high, compared to parents with lower support. The steeper decline among parents with high support suggests that family and friend support may amplify reductions in racial socialization stress as experiences of racism increase. Together, these findings indicate that social support from family and friends serves as a salient contextual resource in shaping parents’ stress responses to racism. Lastly, Figure 4 revealed that parents who reported lower racial discrimination-related stress and higher emotional suppression reported higher racial socialization stress. Conversely, as racial discrimination concerns increased, suppressing unpleasant emotions was associated with lower stress levels compared to parents with high racial discrimination concerns and low emotional suppression.

## 9. Discussion

The current study examines whether perceptions of coping self-efficacy (i.e., problem-focused coping, support from family and friends, and stopping unpleasant emotions) moderated the relationship between being bothered by racism and racial socialization stress among Black American caregivers. Results revealed that being bothered by racial discrimination was associated with lower racial socialization stress. These findings suggests that caregivers who are highly bothered by racial discrimination may recognize racism as a predictable reality and determine racial socialization as necessary and expected, thus reducing the stress of “the talk.” So, in this case, engaging in conversations around race and racism as necessary and expected and feel more psychologically prepared to engage in those conversations. In other words, the affective responses to racial discrimination (i.e., the extent to which they reported being bothered by discriminatory experiences) may decrease Black parental stress around preparation for biased racial socialization by providing a relevant example through which to discuss strategies to navigate racial harm with their children (Thomas & Blackmon, 2015). The interaction effects revealed that feeling more efficacious at seeking support from family and friends and stopping unpleasant emotions were associated with less racial socialization stress when parents were highly bothered by racism. Conversely, feeling efficacious at problem-focused coping was associated with comparatively more racial socialization stress when parents were highly bothered by racism. In the following discussion, we situate each of these findings within the extant literature, acknowledge the strengths and limitations of the current study, and offer implications for research, practice, and policy.

### 9.1. Problem-Focused Coping Self-Efficacy

The first interaction analysis examined whether problem-focused coping self-efficacy moderates the relationship between being highly bothered by racism and racial socialization stress. Findings indicated that feeling highly efficacious about one’s ability to use problem-focused coping was associated with more racial socialization stress when compared to those who felt less efficacious. These findings highlight the limit of individual problem-focused coping in highly bothersome racial contexts, providing further evidence of the goodness-of-fit coping hypothesis which suggests that problem-focused coping is most efficacious when the conditions are highly controllable (Lazarus & Folkman, 1984; Park et al., 2012). Racism may or may not be perceived as a controllable stressor and parents’ general ability to use problem solving may not aid them when they experience racism or when they need to communicate helpful strategies to their children. Further, racial socialization may be a form of familial problem-focused coping response, in which parents prepare Black children and adolescents for racism based on their own experiences (McNeil Smith et al., 2016). For instance, caregivers who engage in active problem-solving may experience greater cognitive engagement with racial stressors, thereby reducing the magnitude of shifts in racial socialization stress across levels of discrimination. Thus, rather than indicating that problem-focused coping buffers stress, this pattern may reflect a stabilization process in which active coping reduces fluctuations in stress responses. Within the sociohistorical stress framework (Murry et al., 2018), such patterns underscore that coping strategies may shape the form of stress response without eliminating the strain. It is also important to note that coping strategies may function differently depending on the controllability of the stressor. It is possible that in contexts where stressors are systemic and enduring, strategies emphasizing direct problem-solving may not uniformly reduce psychological strain. Importantly, these findings do not imply that problem-focused coping is maladaptive; rather, they suggest that its effects on racial socialization stress may differ from traditional buffering models in racialized contexts. Further research is warranted to explore the qualitative meaning of problem-focused coping self-efficacy in the context of racial discrimination and racial socialization stress. In-depth inquiry may help clarify how caregivers interpret and enact problem-solving strategies when navigating racial discrimination and racial socialization messaging.

### 9.2. Support Seeking Self Efficacy

The second interaction analysis found that racial socialization stress was lower for parents who felt they could rely upon their social support networks when they were highly bothered by racism. These findings suggest empirical support for the Black American cultural adage, “It takes a village to raise a child”—wherein Black American parents embedded in supportive kin networks share child rearing burdens. Interestingly, previous studies have largely found that social support is most advantageous at lower levels of stress exposure (Brondolo et al., 2009). Still, social support is a commonly used strategy for navigating racial stress. A systemic review of Black Americans and Canadians coping with racism found that social support was the most frequently used strategy for addressing racial stress in qualitative studies (Jacob et al., 2022). In practice, feeling like one has access to support from family and friends may help to alleviate stress as parents/caregivers realize that both collective challenges and resources are shared. In addition to family and friend support, understanding how these mechanisms help alleviate racial discrimination and racial socialization stress would provide further information on the role that these support systems provide and will provide opportunities for preventive interventions. Other supports should also be considered, such as one’s spiritual and religious beliefs. Further, qualitative and longitudinal approaches may identify whether these supports provide emotional validation, shared coping resources, collective meaning-making, or practical guidance in navigating racialized experiences.

### 9.3. Stopping Unpleasant Emotions Self-Efficacy

In the final interaction analysis, we examined whether one’s perception of their ability to stop unpleasant emotions moderated the relationship between being bothered by racism and racial socialization stress. Findings indicated that those who felt they were more efficacious at stopping unpleasant emotions reported less racial socialization stress when highly bothered by racism when compared to those who did not feel efficacious. This finding may best be conceptualized in terms of one’s feelings about their ability to emotionally regulate or utilize avoidance coping. Although limited, existing studies suggests that emotional regulation may act as either a moderator or mediator between exposure to racism and mental health outcomes (Cole et al., 2024; Graham et al., 2015). In one study examining the relationship between exposure to racism and anxiety in a sample of Black Americans, researchers found that poor emotional regulation skills significantly moderated the relationship between frequency of racist events and anxious arousal such that those with fewer emotional regulation skills were also more likely to report anxious arousal. Notably, there was no significant relationship for those with strong emotional regulation skills—suggesting a buffering effect (Graham et al., 2015). Further, this association between stopping unpleasant emotions and thoughts may provide only short-term emotional relief or allow caregivers to remain composed during difficult conversations. For instance, suppression-based coping has been associated with longer-term psychological costs, including increased physiological arousal and internalized distress (Patel & Patel, 2019). In the context of racism, chronic and structurally embedded stressor, emotional suppression may function as a temporary regulatory strategy rather than a sustainable form of copping. Longitudinal research is needed to determine whether such strategies represent effective regulation or deferred distress in the context of chronic racism. Therefore, the pattern observed in the current study may also reflect deliberate emotional regulations intended to maintain composure during difficult conversations. However, without direct measures of regulatory strategy or longitudinal outcomes, we cannot determine whether these processes represent adaptive regulation or avoidance. Future research is warranted.

### 9.4. Implications

One major implication of the current study is the need to support Black parents. Drawing from Murry et al.’s (2018) Sociohistorical Integrative Model for the Study of Stress in Black Families the impact of stress on parental psychological functioning and subsequent parenting practices has the potential to influence the mental health functioning of the family. Therefore, ensuring that parents are socially supported may help them to navigate the complexities of racially socializing Black American children. The current study demonstrates that parents who report having high levels of friend and family support were less stressed by racial socialization. Practitioners who can support Black parents may further support clients by encouraging them to lean more heavily on their social networks for support, deepen existing relationships, or seek out new friendships based in mutual respect and care. Policy makers may further support these efforts by advocating for and recognizing anti-racist, affirming third spaces to facilitate relationship building among community members. Future research examining support seeking among Black American caregivers might utilize different methods, such as social network analysis to determine the quality and utility of relationships within Black American kin networks.

The current study also suggests that feeling like one can stop unpleasant emotions may be an important aspect of coping when highly bothered by racism. Implications for practitioners suggest the need to utilize clinical treatment tools such as EMBRace (Anderson et al., 2018a) that aid parents in learning emotional regulation skills they can model and teach to their children to use in racially challenging situations. EMBRace has shown early promise in helping contemporary parents deepen their own socio-emotional development with implications for further facilitating parental psychological functioning and promotive parenting practices. Policymakers seeking to support the emotional regulation of Black parents might support initiatives that help to relieve parenting burdens. For example, supporting policies that allow for paid mental health days for employees or encourage screening patients regarding their coping skills in clinical settings may serve to support parents.

A significant implication of the present study underscores the paramount importance of sustained efforts to eradicate racism and other oppressive systems. Policymakers are tasked with the complex task of navigating an increasingly hostile policy environment. Therefore, advocating for policy changes that benefit Black Americans may require more fortitude, innovation, and cross-sector collaboration than recent eras. It may be helpful to engage with local and state politics until the federal policy environment becomes more amenable to equity-minded initiatives. Policies may be more likely to be supported further when they are framed through the lens of interest convergence or rather when dominant group members can be persuaded that equity can benefit them.

### 9.5. Strengths and Limitations

A strength of the current study is in its novelty in considering how coping self-efficacy moderates the relationship between subjective parental experiences of being bothered by racism and self-reported racial socialization stress. The finding that problem-focused coping self-efficacy was associated with greater racial socialization related stress is particularly novel as it suggests that feeling effective at solving problems generally does not always translate into coping with all racialized stressors. Although there are many strengths of the current study, it is not without its methodological limitations. First, we analyzed only one wave of data. This limited our ability to determine causation between the variables. Thus, future studies may employ longitudinal methods to better understand how temporality influences the relationship between these variables. Second, while the measure we used to examine coping self-efficacy proved useful, we did not specifically ask how parents in the sample coped with racism specifically. As self-efficacy is largely domain specific, one’s perception of their ability to cope with daily stressors may not be indicative of their ability to navigate and overcome racial stressors. Therefore, future studies may examine racialized coping self-efficacy specifically to better understand how Black parents appraise their ability to cope with racial stressors. An additional concern was the low alpha of the family and friend support scale (α = 0.66). We note this as a limitation and interpret findings involving this moderator with caution.

It is also crucial to consider the context. Our sample was primarily Black caregivers from urban and suburban settings. Rural Black communities, which were geographically distant from the recruitment sites (approximately 3 to 4 h away), were not represented in this sample, potentially limiting generalizability to more rural caregiving contexts. Also, caregivers who were more engaged with racial issues or more comfortable discussing discrimination may have been more likely to participate. This potential selection bias should be considered when interpreting the findings. Lastly, although the sample included both mothers and fathers, we did not examine potential differences across caregiver gender roles. Prior research suggests that mothers and fathers may engage in racial socialization differently (Caldwell et al., 2026; Smith-Bynum et al., 2016); therefore, future studies should explore whether coping processes and racial socialization stress vary by caregiver role.

## 10. Conclusions

Overall, the findings of the study corroborate previous studies in documenting the impact of racial discrimination on parenting stress, particularly as it relates to Black families engaging in conversations about race and racism with their children. Another significant finding in this study was the role of coping self-efficacy, more specifically, stopping unpleasant thoughts and emotions, using problem-focused coping strategies, and seeking support from family and friends. The findings underscored the varied ways that coping self-efficacy is utilized and the way that it operates in the context of race-related stress. Moving forward, it is important to continue to understand the multitude of ways that parents are using their internal processes to address racial discrimination experiences and engaging in conversations with their children about race and racism. Our findings contributed to this literature by noting the unique role of specific coping self-efficacy strategies in the context of race-related stress. Further, longitudinal research is warranted to examine how problem-solving and stopping unpleasant thoughts and emotions may carry long-term psychological or relational consequences, particularly when enacted in response to stressors such as racism. That said, given the cross-sectional design, these findings should be interpreted cautiously. Moving forward, understanding and being intentional about coping self-efficacy and how Black parents engage in strategies that promote or inhibit positive developmental competencies will further help in developing interventions and creating safe spaces to learn about other coping strategies that are used when parents are encountering racism. Furthermore, future research should explore how culturally specific protective factors and moderators can attenuate the influence of racial discrimination on parenting stress and psychological distress within larger samples of caregiver-child dyads (Willis et al., 2024). By foregrounding the complexity of coping self-efficacy strategies, this study contributes to existing scholarship on racial stress and coping among Black American caregivers.

## Figures and Tables

**Table 1:** Means and Standard Deviations.

	*M*	*SD*
Age	37.29	3.64
Education	4.29	0.699
Income	2.20	0.514
Number of Children	2.09	0.737
Racial Socialization Stress	3.45	0.735
Racial Discrimination	3.88	0.535
Problem-Focused Coping	6.87	0.747
Stop Unpleasant Thoughts & Emotions	6.88	0.794
Support from Family & Friends	6.88	0.875

Note. Education = Associate’s degree or some college; income = $35,000 to $50,000 per year; number of Children = 2.

**Table 2:** Correlations.

Variables	1	2	3	4	5	6	7	8
1. Age								
2. Education	−0.15 **							
3. Income	−0.07	0.23 **						
4. Number of Children	0.31 **	0.28 **	−0.23 **					
5. Racial Discrimination	−0.18 *	−0.17 **	0.38 **	−0.49 **				
6. Unpleasant Thoughts	−0.14 **	0.16 **	0.28 **	−0.13 **	0.29 **			
7. Problem-Focused Coping	−0.05	0.28 **	0.24 **	0.02	0.11 **	0.78 **		
8. Support from Family & Friends	−0.09 *	0.17 *	0.21 **	−0.04	0.17 **	0.68 **	0.69 **	
9. Racial Socialization Stress	0.16 **	0.15 **	−0.45 **	0.42 **	−0.65 **	−0.40 **	−0.27 **	−0.30 **

Note. *p* < 0.05 *, *p* < 0.010 **.

**Table 3:** Hierarchical Regression for Racism, Coping and Stress.

	*B* (*SE*)	*β*	*p*
**Model 1**			
Age	0.01 (0.01)	0.01	0.656
Education	−0.44 (0.04)	−0.42	0.001 ***
Number of Children	0.26 (0.04)	0.26	0.001 ***
Income	0.24 (0.05)	0.16	0.001 ***
Gender (1 = Mothers, 0 = Fathers/Stepfathers)	−0.06 (0.05)	−0.04	0.201
**Model 2**			
Age	0.01 (0.01)	0.01	0.843
Education	−0.27 (0.03)	−0.26	0.001 ***
Number of Children	0.08 (0.03)	0.08	0.013 *
Income	0.14 (0.04)	0.09	0.001 ***
Gender (1 = Mothers, 0 = Fathers/Stepfathers)	−0.04 (0.04)	−0.02	0.319
Racial Discrimination	−0.66 (0.04)	−0.49	0.001 ***
**Model 3**			
Age	−0.05 (0.01)	−0.02	0.386
Education	−0.24 (0.03)	−0.22	0.001 ***
Number of Children	0.09 (0.03)	0.09	0.004 **
Income	0.22 (0.04)	0.15	0.001 ***
Gender (1 = Mothers, 0 = Fathers/Stepfathers)	−0.09 (0.04)	−0.06	0.019 *
Racial Discrimination	−0.58 (0.04)	−0.43	0.001 ***
Problem-Focused Coping	−0.06 (0.04)	−0.06	0.130
Stop Unpleasant Thoughts & Emotions	−0.11 (0.04)	−0.12	0.005 **
Support from Family & Friends	−0.07 (0.03)	−0.08	0.029 *
**Model 4**			
Age	−0.08 (0.01)	−0.38	0.155
Education	−0.22 (0.03)	−0.21	0.001 ***
Number of Children	0.09 (0.03)	0.09	0.002 **
Income	0.20 (0.04)	0.14	0.001 ***
Gender (1 = Mothers, 0 = Fathers/Stepfathers)	−0.08 (0.04)	−0.05	0.029 *
Racial Discrimination	−0.57 (0.04)	−0.43	0.001 ***
Problem-Focused Coping	−0.09 (0.04)	−0.09	0.028 *
Stop Unpleasant Thoughts & Emotions	−0.10 (0.04)	−0.10	0.014 *
Support from Family & Friends	−0.04 (0.03)	−0.05	0.162
Racism × Problem-Focused Coping	0.40 (0.07)	0.23	0.001 ***
Racism × Stop Unpleasant Thoughts & Emotion	−0.12 (0.05)	−0.07	0.026 *
Racism × Support from Family & Friends	−0.37 (0.06)	−0.20	0.001 ***

Note. *p* < 0.05 *, *p* < 0.01 **, *p* < 0.001 ***.

## Data Availability

Dataset available on request from the authors.
